# Parkinson’s disease motor intervention patterns: a network meta-analysis based on patient motor function

**DOI:** 10.3389/fneur.2024.1432256

**Published:** 2024-09-05

**Authors:** Hongfei Zhao, Li Zhang, Jingling Yang, Wanru Guo, Chunyang Sun, Runbo Shi, Zhen Wang

**Affiliations:** ^1^Wushu College, Shanghai Sport University, Shanghai, China; ^2^Xiamen Medical College, Xiamen, Fujian, China; ^3^Yueyang Hospital of Integrated Chinese and Western Medicine, Shanghai University of Traditional Chinese Medicine, Shanghai, China; ^4^School of Exercise and Health, Shanghai Sport University, Shanghai, China

**Keywords:** network meta-analysis, Parkinson’s disease, exercise intervention, motor function, randomized controlled trial

## Abstract

**Background:**

Parkinson’s disease is characterized by symptoms such as bradykinesia and rigidity, which worsen as the disease progresses, significantly impacting patients’ independence and quality of life. This study utilizes a network meta-analysis approach to quantify information gathered from randomized controlled trials (RCTs) regarding motor interventions that effectively improve the motor function of Parkinson’s disease patients, aiming to provide evidence for selecting appropriate exercise intervention strategies for patients.

**Methods:**

A systematic search strategy for randomized controlled trials (RCTs) restricted to English was constructed based on multiple biomedical databases. Databases searched included PubMed, Embase, Cochrane, Web of Science, CINAHL, CBM, China National Knowledge Infrastructure (CNKI), Wan fang, VIP, etc., with searches conducted from inception to July 9, 2023. Two authors screened all studies, extracted data, and used frequency domain analysis methods. Network meta-analysis was performed using STATA software version 18.0 to compare and rank exercises that could effectively improve the motor function of Parkinson’s disease patients (measured by indicators such as MDS-UPDRS-III, TUG, BBS, Mini-BES Test, 6MWT scores). Additionally, a series of analyses and evaluations were conducted, such as assessing the methodological quality of included studies using the Cochrane risk of bias tool.

**Results:**

The network meta-analysis included a total of 111 studies involving 5,358 participants, 133 intervention experiments, and 31 intervention measures. Although most exercise interventions showed effectiveness, cumulative ranking curves under the surface (SUCRA) values showed that archery exercise significantly improved patients’ MDS-UPDRS-III scores (SUCRA = 95.6%), significantly superior to routine care [standardized mean difference (SMD = 16.92, 95%CI = −28.97, −4.87)]. High-intensity and agility exercise (High strength and agility) referred to as high-intensity exercise or agility training or a combination of both, collectively termed as high-intensity agility training, significantly improved patients’ completion time for the time-up-and-go test (SUCRA = 99.7%), (SMD = −7.88, 95%CI = −9.47, −6.28). Dance and Tai Chi exercises significantly improved patients’ balance abilities: Mini-Balance Evaluation Systems Test (SUCRA = 77.9%), (SMD = 5.25, 95%CI = −0.42, 10.92) for dance intervention and Berg Balance Scale (SUCRA = 94.7%), (SMD = 11.22, 95%CI = 3.26, 19.18) for Tai Chi intervention. Dance also significantly improved patients’ walking ability in the 6-min walk test (SUCRA = 80.5%), (SMD = 71.31, 95%CI = 13.77, 128.84).

**Conclusion:**

Compared to other exercises, archery, dance, Tai Chi, and high-intensity agility exercises demonstrate superior efficacy in improving the motor function of Parkinson’s disease patients.

## 1 Introduction

Parkinson’s Disease (PD) is a common neurodegenerative disorder characterized by the degeneration and death of dopaminergic neurons. It arises from the slow loss of midbrain cells in the substantia nigra and is the second most prevalent neurodegenerative disease after Alzheimer’s disease. With the global population aging, the number and proportion of individuals aged 65 and above are rapidly increasing, leading to an unprecedented rise in the number of Parkinson’s disease patients (1). It is estimated that by 2040, the global number of Parkinson’s disease patients will exceed 12 million (2). Parkinson’s disease not only affects the elderly; many individuals under 50 years old are also developing the condition, with no one immune to it (3). The typical pathology of Parkinson’s disease includes bradykinesia, rigidity, reduced movement amplitude, and decreased automatism (4). Moreover, patients’ functional abilities are impaired, increasing the risk of falls and related injuries as the disease progresses (5). This constitutes a significant burden on patients’ independence and quality of life. While dopaminergic medications can improve certain aspects of patients’ motor abilities, such as walking speed and stride length, they face multiple challenges and may not significantly alter temporal features and intermittent symptoms (6, 7). For some patients, medication interventions may even exacerbate motor dysfunction and impair mobility, including fluctuations in motor response (so-called wearing-off) and inducing movement disorders.

To date, there is no cure for PD. Dopaminergic drugs are the mainstay of treatment (8), alleviating motor symptoms in PD patients. However, they have limitations; prolonged use can lead to drug resistance, and some patients may experience hallucinations, visual illusions, auditory hallucinations, impulsivity, and compulsive behaviors (9). Complications resulting from long-term levodopa therapy and reduced dosage of dopamine agonists (10). Moreover, medications also have certain toxic side effects (11). Surgical deep brain stimulation (DBS) treatment may accelerate the initiation of PD patients’ responses and improve active inhibition of functional disorders. However, DBS treatment carries risks of infection and rejection reactions, surgical contraindications, and cannot prevent or alleviate the appearance of non-motor symptoms and the progression of the disease.

However, exercise, along with rehabilitation therapy, psychological intervention, and caregiving, is applicable throughout the course of Parkinson’s disease treatment. It is an important adjunct therapy that is convenient, economical, and suitable for long-term adherence. Compared to medication, exercise therapy may have better effects on patients’ motor symptoms. This evidence is supported by cross-sectional studies, longitudinal observational studies, and prospective intervention trials (12–16). However, there are also few studies indicating that although the exercise duration is similar, there is no significant improvement in patients’ motor abilities. This difference may be due to differences in cognitive involvement, and the severity of the disease may also affect the outcomes, leading to discrepancies (17, 18).

Recently, two network meta-analyses were published, examining evidence of different exercise interventions to improve patients’ motor abilities. The first study included 60 randomized controlled trials with a total sample size of 3,537, analyzing evidence of 19 exercises for improving postural balance in Parkinson’s disease patients. The network meta-analysis results showed that exercise significantly improved patients’ time-up-and-go time. Dance significantly improved patients’ Berg balance scale scores, and rhythmic auditory stimulation significantly improved patients’ Mini-Balance Evaluation Systems Test scores. However, this study’s classification of exercise interventions was relatively broad and unclear, only analyzing evidence of certain categories of exercise improving patients’ motor symptoms, without providing detailed intervention methods and duration recommendations for patients. The second study included 20 trials with 719 patients, focusing on aerobic and resistance exercises’ evidence of intervention in Parkinson’s disease patients’ motor abilities. The indicators used in the article mainly focused on patients’ walking abilities, such as 6MWT, 10TWM, TUG, which may lead to some one-sidedness in analyzing the improvement of patients’ motor abilities.

In this systematic review, we adopted a network meta-analysis method to simultaneously compare multiple treatments in a single analysis by combining direct and indirect evidence from randomized controlled trials (RCTs) within the network. Traditional meta-analysis can only collect studies designed for direct comparison of intervention measures.

Network meta-analysis (NMA) is a universal technique used to compare multiple intervention measures simultaneously (e.g., A vs. B, B vs. C) (19). NMA can compare multiple intervention measures by combining direct and indirect comparisons, thereby selecting the best intervention measures based on the relative effectiveness of different intervention measures in the evidence network (20).

Here, we aim to evaluate various exercise interventions through network meta-analysis (NMA), including treadmill training, strategy training, aerobic exercise, aquatic exercise, balance and gait training, dual-task training, boxing, dance (such as Irish dance, Sardinian dance, square dance, partner dance, different rhythmic dance therapies), fitness qigong (such as Eight Pieces of Brocade, Five Animals Frolic, Six Word Formula), Tai Chi, mindfulness meditation, resistance exercise (such as weightlifting, resistance band exercise, progressive resistance exercise), high, medium, and low-intensity exercises, sports games, rock climbing, and other exercise interventions related to Parkinson’s disease randomized controlled trials (RCTs).

Although many systematic reviews and meta-analyses have discussed the effectiveness of various physical therapies in PD, each has only compared two or several treatment methods. Most reviews and meta-analyses include non-randomized controlled trials or lack quantitative analysis, comparing non-drug, physical interventions with placebos, waiting lists, or routine treatments, lacking comprehensive, systematic, and detailed evidence and corresponding precise outcome indicators to illustrate the relative effects of all tested exercises on patients’ motor abilities. This lack of integration is important because different exercise interventions vary in cost and effectiveness. This study attempts to systematically review previous randomized controlled trials, refine various exercise interventions into different types for reevaluation, and compare and rank exercises that can effectively improve Parkinson’s disease patients’ motor abilities, providing further evidence for clinicians and patients in selecting appropriate exercise intervention methods and duration.

## 2 Methods

### 2.1 Eligibility criteria and study search

Following the Preferred Reporting Items for Systematic Reviews and Meta-Analyses Extension for Network Meta-Analyses guidelines (21), we conducted searches in the following databases from inception to July 9, 2023, for randomized controlled trials (RCTs) related to medical interventions for Parkinson’s disease: PubMed, Embase, Cochrane, Web of Science, CINAHL, CBM, China National Knowledge Infrastructure (CNKI), Wanfang, and VIP databases. We used a combination of medical subject headings (MeSH terms or Emtree terms) and free text related to Parkinson’s disease, exercise interventions, and randomized controlled trials, including: (1) MeSH terms: Parkinson’s disease, free text: Parkinson’s disease, idiopathic Parkinson’s disease, Lewy body Parkinson’s disease, primary Parkinson’s disease, tremor-predominant Parkinson’s disease; (2) MeSH terms: Exercise intervention, free text: aerobic exercise, resistance training, strength training, balance exercises, balance, dual-task training, stretching exercises, Tai Chi, Five Animals Frolic, Eight Pieces of Brocade, qigong, yoga, dance, boxing, resistance training, aquatic exercise; (3) MeSH terms: randomized controlled trials, random control, placebo. MeSH and free words within each group were linked with “OR,” and each group was searched with “AND.”

### 2.2 Study selection criteria

#### 2.2.1 Inclusion criteria

(P) Participants: human subjects with early to mid-stage PD classified according to the Hoehn and Yahr (H&Y) scale (I–III), with an average age ≥66 years; (I) Intervention: Exercise training; (C) Comparator: randomized control group; (O) Outcomes: unified Parkinson’s Disease Rating Scale (MDS-UPDRS-III), Time Up and Go test (TUG), Mini-Balance Evaluation Systems Test (Mini BES Test), Berg Balance Score (BBS), 6-min walk test (6MWT); (S) Study design: English-language and published RCTs.

#### 2.2.2 Exclusion criteria

(1) Studies involving participants with other neurological diseases; (2) Studies with incomplete data or unable to obtain statistical analysis; (3) Studies using outcome measurements other than MDS-UPDRS part III, TUG, 6MWT, BBS, Mini BES Test, e.g., original UPDRS or parts 1, 2, or 4; (4) Studies without control groups or involving only single acute training protocols, abstracts, or conference poster presentations; (5) Studies from non-randomized controlled trials (reviews, comments, animal studies).

### 2.3 Study selection

After excluding and identifying eligible literature, all relevant literature was stored in the EndNote 20 reference manager. Two authors independently screened the titles, abstracts, and full texts of potentially eligible RCTs, with discrepancies resolved by consensus. Data extraction was completed by the primary author, including participant characteristics such as sample size, age (years ± SD), disease duration (years ± SD), H&Y stage (mean ± SD), MDS-UPDRS part III score (mean ± SD), medication status during the trial (ON or OFF), type, frequency, and duration of exercise intervention. Additionally, data from ongoing or forthcoming trials were retrieved from the ClinicalTrials.gov and Chinese Clinical Trial Registry platforms. Gray literature was also considered in the search. Manual searches of the reference lists of included literature and relevant articles were conducted to identify eligible studies. Risk of bias (RoB) was assessed using the revised Cochrane rct Collaboration tool (22), with assessments made across six domains: (1) bias arising from the randomization process, (2) deviations from intended interventions, (3) missing outcome data, (4) outcome measurement, (5) selection of reported results, and (6) overall bias. Disagreements were resolved through consensus, and a risk of bias table was created. Risk assessments for each trial were independently entered into Review Manager (RevMan 5.4), generating a summary of bias risk alongside meta-analysis results.

### 2.4 Statistical analysis

Network analysis was conducted using STATA 18.0 and analyzed using a Frequentist framework following the PRISMA NMA guidelines. For all eligible trials, mean and standard deviation post-intervention values were compared. To reveal all available effects for each exercise intervention, a network evidence graph was generated as a simple summary description. In the network graph, nodes represent various exercise interventions, with node size proportional to the number of study participants. The connecting edges display direct pairwise comparisons, with line thickness correlated to effect size.

Surface under the cumulative ranking (SUCRA) plots provide a simple numerical summary of the cumulative ranking probabilities for each intervention, serving as an estimate of the probability used to rate exercise interventions (23). A higher SUCRA value indicates a higher likelihood of an exercise intervention being at the top level or extremely effective, while a lower value indicates the intervention is likely the worst. We examined global consistency, fitting inconsistent and consistent models, and used node-splitting analysis models to determine local consistency. *p* > 0.05 indicates no significant inconsistency between direct and indirect comparisons, adopting a consistency model; otherwise, an inconsistency model is used. To detect publication bias, a funnel plot was created as a concise description, such as publication bias, selective reporting, or other biases (24).

## 3 Results

### 3.1 Study identification

**Figure 1:**
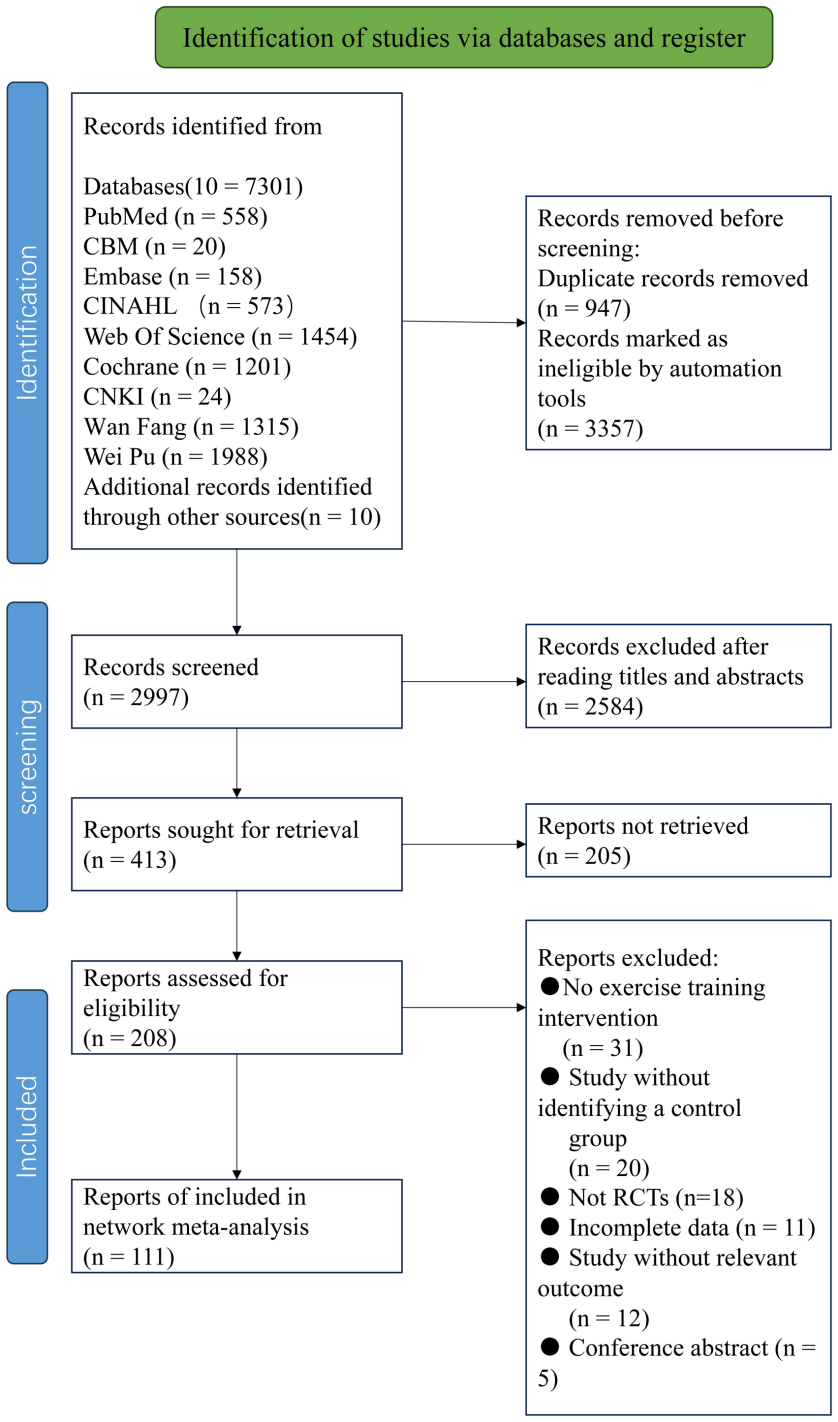
The process of selection of the eligible studies.

According to the pre-defined search strategy, a total of 7,301 articles were retrieved. After excluding duplicates and for other reasons, 2,997 articles remained, which were screened based on titles and abstracts. Then, 2,584 articles were excluded as irrelevant literature. A total of 413 results were confirmed by reviewing full texts, and 302 articles were subsequently excluded (reasons including non-randomized controlled trials, incomplete data, conference papers, inconsistency with intervention measures, etc.). Finally, this study included a total of 111 articles (Figure 1).

### 3.2 Study characteristics

Table 1 presents the characteristics of 111 eligible randomized controlled trials published between 2002 and 2023, involving 5,358 participants. In this network meta-analysis (NMA), there were a total of 133 intervention experiments and 31 intervention measures, such as GE: Gait Exercise (gait posture intervention, aerobic walking); RC: Routine Care; RE: Resistance Exercise (weightlifting, resistance band, strength training interventions, etc.); BE: Balance Exercise (stability exercises, balance training); HT: Hydrotherapy Exercise (aquatic exercise); AH: Archery Exercise; ST: Stretching Training (limb stretching, joint stretching); TC: Tai Chi; NW: Nordic Walking; YG: Yoga; TTC: Treadmill Training (high-intensity, moderate-intensity, low-intensity treadmill interventions); TB: Treadmill Balance (treadmill combined with balance intervention); DA: Dance (tango, Irish dance, improvisational dance, waltz); SG: Sports Games (VR, active video games); MM: Mindfulness Meditation; DTT: Dual-Task Training; FQ: Fitness Qigong (Six-word Secret, Five Animals Frolic, Eight Pieces of Brocade); HAS: High-intensity Agility Training; SC: Sport Climbing; SST: Sports Strategy Training; Da2: Binary Rhythm Dance; BO: Boxing; MIT: Moderate Intensity Training; STT: Sensory Attention Training; ESP: Elastic Band Pilates; RAB: Cycling; AE: Aerobic Exercise; WLR: Weightlifting Resistance Exercise; EAR: Elastic Band Resistance Training; CD: Couple Dance; BK: Kickboxing. In the included studies, most exercise interventions were compared with routine care, stretching exercises, and aerobic exercises. Among all eligible studies, 90 randomized controlled trials (25–77) were two-arm trials, while 21 randomized controlled trials (16, 78–93) were three-arm trials. The duration of exercise intervention in the included trials ranged from 4 to 96 weeks (mean duration 14.5 weeks, SD 15.3), with the total number of intervention sessions ranging from 6 to 288 (mean 32.41 sessions, SD 30.19), weekly exercise intervention frequency ranging from 1 to 5 times (mean frequency 2.5, SD 1.05), and individual session duration ranging from 15 to 120 min (mean duration 53.25 min, SD 21.81).

**Table 1:** Characteristics of the included studies.

Characteristics	Mean	SD
Age	66.5	7.81
Exercise period (weeks)	12.9	13.1
Number of interventions (frequency)	32.4	30.1
Practice time (minutes)	53.2	21.8
Hoehn and Yahr (H&Y)	2.16	0.36

### 3.3 Quality assessment

**Figure 2:**
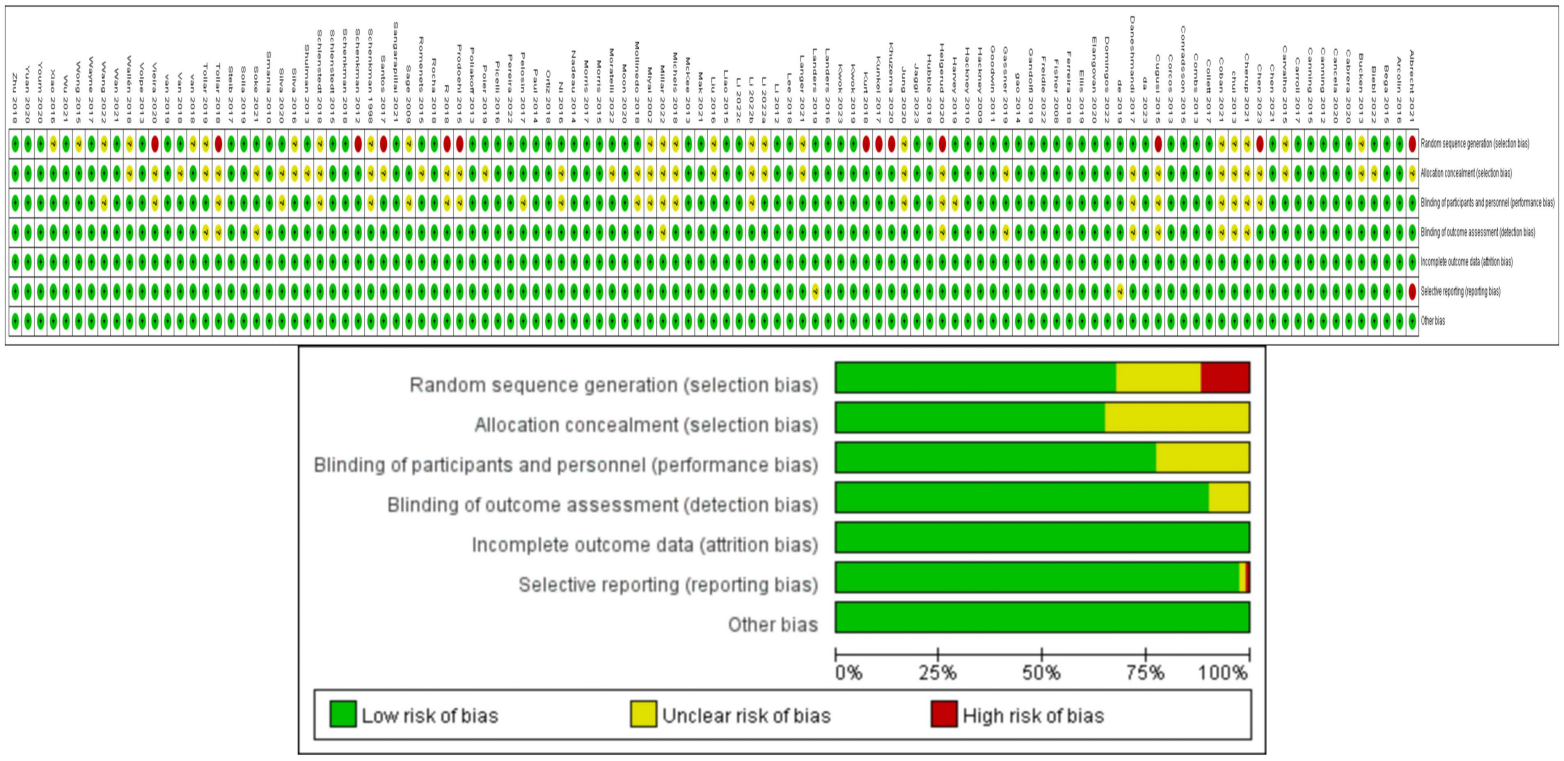
Analysis of the risk of bias in accordance with the Cochrane collaboration guideline.

The methodological quality assessment of eligible randomized controlled trials is shown in Figure 2, indicating an overall high quality of the included literature. Thirteen trials had notable flaws in randomization, and blinding was not clearly described in one trial, leading to incomplete reporting of trial results and consequently categorized as medium risk. In 23 trials, randomization and blinding were mentioned but not elaborated upon, thus classified as low risk.

### 3.4 Network analysis

#### 3.4.1 MDS unified-Parkinson disease rating scale

**Figure 3:**
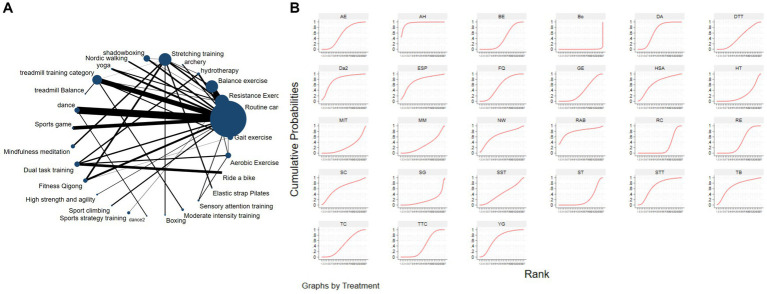
The NMA figure for MDS-UPDRS-III **(A)**. The SUCRA plot for MDS-UPDRS-III **(B)**. GE, gait exercise; RC, routine care; RE, resistance exercise; BE, balance exercise; HT, hydrotherapy; AH, archery; ST, stretching training; TC, shadowboxing; NW, Nordic walking; YG, yoga; TTC, treadmill training category; TB, treadmill balance; DA, dance; SG, sports game; MM, mindfulness meditation; DTT, dual task training; FQ, fitness qigong; HAS, high strength and agility; SC, sport climbing; SST, sports strategy training; Da2, dance2 (Duality Rhythm Dance); Bo, boxing; MIT, moderate intensity training; STT, sensory attention training; ESP, elastic strap pilates; RAB, ride a bike; AE, aerobic exercise.

Figure 3A depicts the network diagram of various exercise interventions for Parkinson’s disease (PD) based on MDS-UPDRS-III scores, indicating that comparisons between DA (dance), ST (stretching training), BE (balance exercise), RE (resistance exercise), and RC (routine care) are common and considered prominent interventions currently. Network meta-analysis (NMA) was conducted to compare the effects of different interventions on the motor abilities of PD patients. Figure 3B and Table 2 show the cumulative ranking curve (SUCRA) of different interventions on motor abilities and pairwise comparisons of 27 exercise interventions for improving motor abilities in PD patients. In SUCRA, different exercise interventions are ranked in terms of the probability of improving patients’ motor abilities (reducing MDS-UPDRS-III scores). As shown in Figure 1B, AH (Archery) ranks first in SUCRA with a value of 96.3%. The rankings are as follows: AH: Archery (SUCRA = 96.3%), Da2: Duality Rhythm Dance (SUCRA = 82.2%), RAB: Ride a bike (SUCRA = 80.4%), ESP: Elastic strap Pilates (SUCRA = 76.3%), YG: yoga (SUCRA = 75.8%), TB: treadmill Balance (SUCRA = 74.0%), STT: Sensory attention training (SUCRA = 71.4%), HAS: High strength and agility (SUCRA = 68.4%), NW: Nordic walking (SUCRA = 66.7%), BE: Balance exercise (SUCRA = 38.9%), RE: Resistance Exercise (SUCRA = 35.3%). These exercise interventions show higher effectiveness in reducing MDS-UPDRS-III scores compared to ST: stretching training (SUCRA = 17.6%) and RC: Routine care (SUCRA = 22.7%). AH: Archery significantly outperforms the control group RC: Routine care (SMD = −18.51, CI = −28.96, −8.06), and also significantly outperforms ST (SMD = −19.59, CI = −30.60, −8.57), SG: Sports game (SMD = −22.33, CI = −36.36, −8.30), AE: Aerobic Exercise (SMD = −14.44, CI = −25.55, −3.34), and others, indicating significant improvement in motor abilities with AH: Archery for Parkinson’s disease. However, due to minimal improvement in motor abilities for PD patients with the BO: Boing intervention, with MDS-UPDRS-III scores increasing rather than decreasing after the intervention (63), BO: Boing ranks last in the probability ranking (SUCRA = 0.4%), indicating inferior effectiveness compared to other exercises. For consistency testing, a fitted inconsistency model yielded *P* > *F* = 0.2295, indicating no significant inconsistency (*p* > 0.05), confirming a consistency model. Furthermore, through node-splitting analysis, both indirect and direct comparison *p*-values were greater than 0.05, indicating good model convergence and favorable iteration effects of the included studies.

**Table 2:** Relative effect sizes of efficacy on MDS-UPDRS-III according to network meta-analysis.

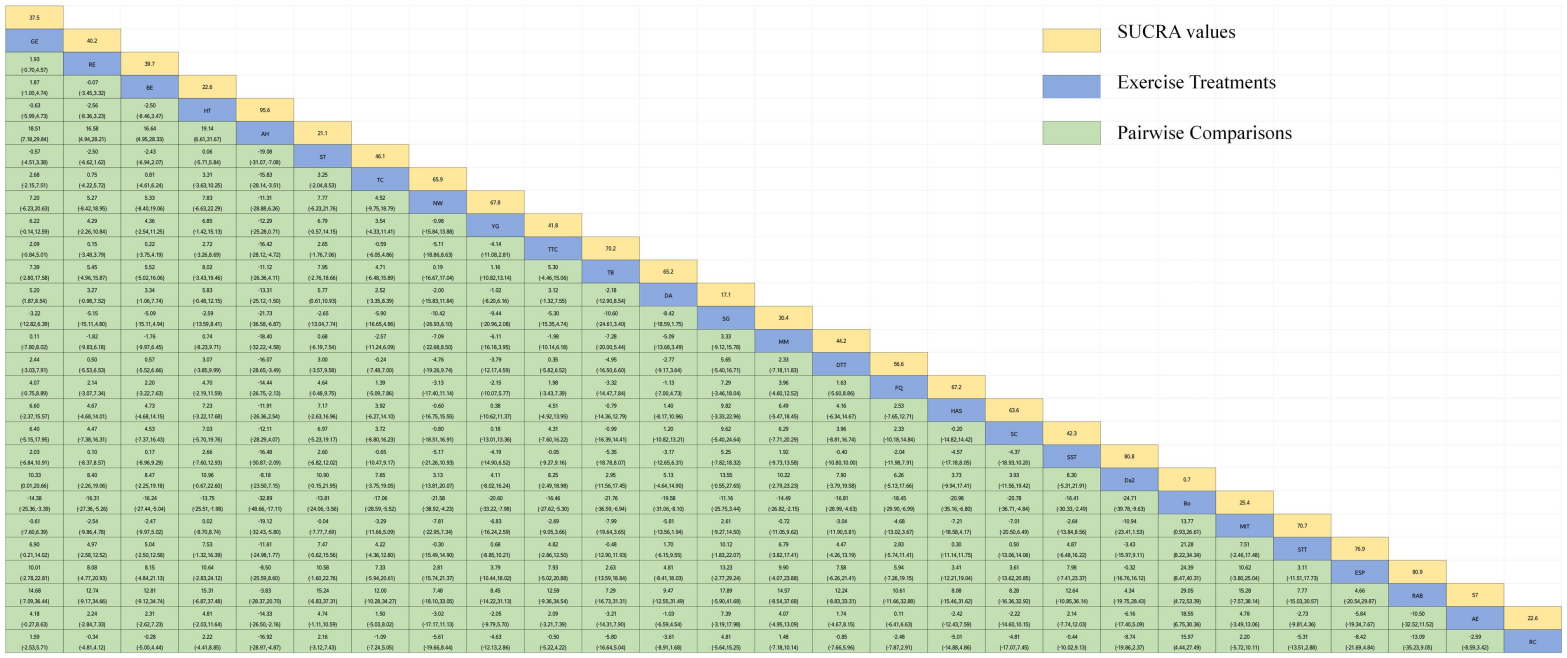

GE, gait exercise; RC, routine care; RE, resistance exercise; BE, balance exercise; HT, hydrotherapy; AH, archery; ST, stretching training; TC, shadowboxing; NW, Nordic walking; YG, yoga; TTC, treadmill training category; TB, treadmill balance; DA, dance; SG, sports game; MM, mindfulness meditation; DTT, dual task training; FQ, fitness qigong; HAS, high strength and agility; SC, sport climbing; SST, sports strategy training; Da2, dance 2 (Duality Rhythm Dance); Bo, boxing; MIT, moderate intensity training; STT, sensory attention training; ESP, elastic strap pilates; RAB, ride a bike; AE, aerobic exercise. The green box is pair-to-pair comparison, the blue box is intervention name, and the yellow box is SUCRA values.

#### 3.4.2 Time up and go test

**Figure 4:**
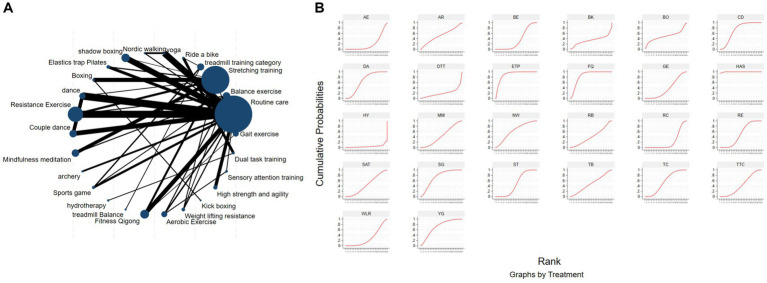
The NMA figure for TUG **(A)**. The SUCRA plot for TUG **(B)**. GE, gait exercise; RC, routine care; BE, balance exercise; ST, stretching training; TTC, treadmill training category; RAB, ride a bike; YG, yoga; NW, Nordic walking; TC, shadowboxing, ESP, elastic strap pilates; Bo, boxing; DA, dance; RE, resistance exercise; CD, couple dance; MM, mindfulness meditation; AH, archery; SG, sports game; HT, hydrotherapy, TB, treadmill Balance; FQ, fitness qigong; AE, aerobic exercise; WLR, weight lifting resistance; BK, kick boxing; HAS, high strength and agility; STT, sensory attention training; DTT, dual task training.

**Figure 5:**
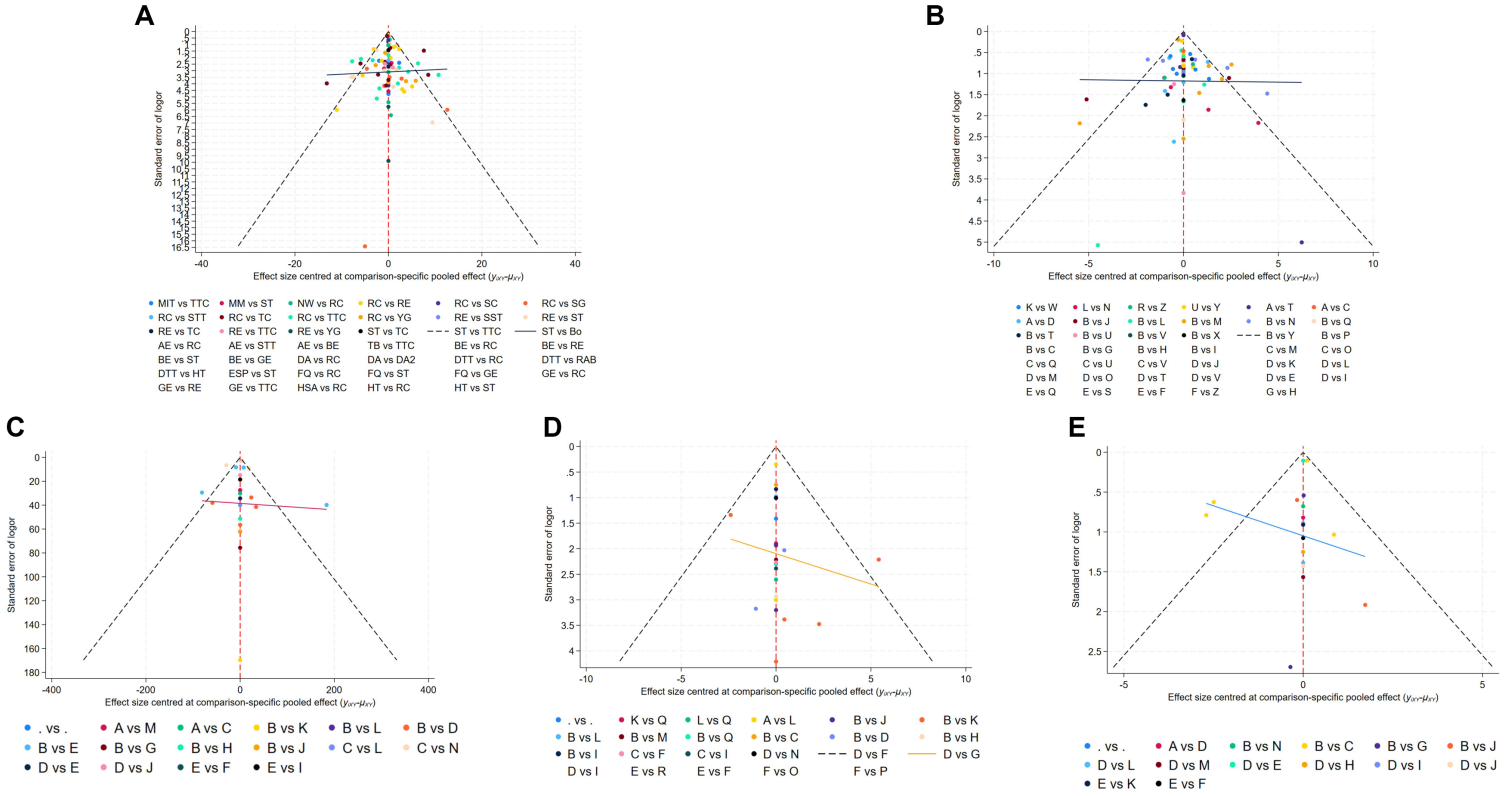
Funnel plot on publication bias of MDS-UPDRS-III **(A)**, TUG **(B)**, 6MWT **(C)**, BBS **(D)**, and Mini BES Test **(E)**.

The NMA of TUG is illustrated in Figure 4A, showing the probability rankings of different exercise interventions in improving TUG time. High strength and agility rank first in SUCRA, as depicted in Figure 4B. The network meta-analysis results indicate that HAS: High strength and agility (SUCRA = 99.7%), ETP: Elastics strap Pilates (SUCRA = 89.1%), FQ: Fitness Qigong (SUCRA = 84.5%), SG: Sports game (SUCRA = 73.2%), CD: Couple dance (SUCRA = 71.4%), YG: yoga (SUCRA = 71.0%), DA: Dance (SUCRA = 65.1%), NW: Nordic walking (SUCRA = 62.7%), TC: shadow boxing (SUCRA = 55.4%), AR: Archery (SUCRA = 51.9%), RE: Resistance Exercise (SUCRA = 50.0%), TB: Treadmill Balance (SUCRA = 48.2%) have lower TUG times compared to the control group RC: Routine care (SUCRA = 21.3%). HAS: High strength and agility significantly outperforms the control group (RC), providing potential effect estimates (SMD = −7.88, CI = −9.47, −6.28). The relative effect sizes of TUG efficacy are shown in Table 3. For consistency testing, a fitted inconsistency model yielded *P* > *F* = 0.9873, indicating no significant inconsistency (*p* > 0.05), confirming a consistency model. Furthermore, through node-splitting analysis, both indirect and direct comparison *p*-values were greater than 0.05, indicating good model convergence and favorable iteration effects of the included studies. See Figure 5 for details.

**Table 3:** Relative effect sizes of efficacy on TUG, according to network meta-analysis.

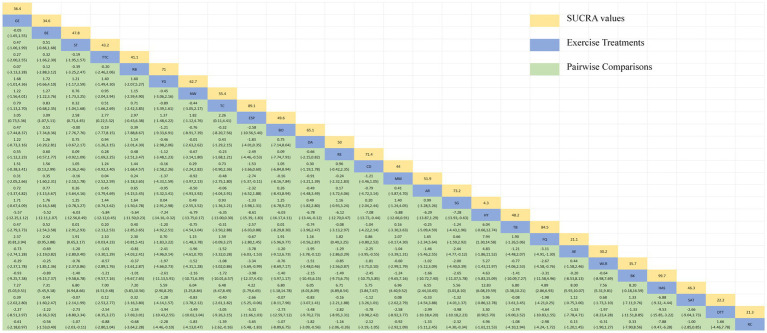

GE, gait exercise; RC, routine care; BE, balance exercise; ST, stretching training; TTC, treadmill training category; RAB, ride a bike; YG, yoga; NW, Nordic walking; TC, shadowboxing; ESP, elastic strap pilates; Bo, boxing; DA, dance; RE, resistance exercise; CD, couple dance; MM, mindfulness meditation; AH, archery; SG, sports game; HT hydrotherapy; TB, treadmill balance; FQ, fitness qigong; AE, aerobic exercise; WLR, weight lifting resistance; BK, kick boxing; HAS, high strength and agility; STT, sensory attention training; DTT, dual task training. The green box is pair-to-pair comparison, the blue box is intervention name, and the yellow box is SUCRA values.

#### 3.4.3 Berg balance scale

**Figure 6:**
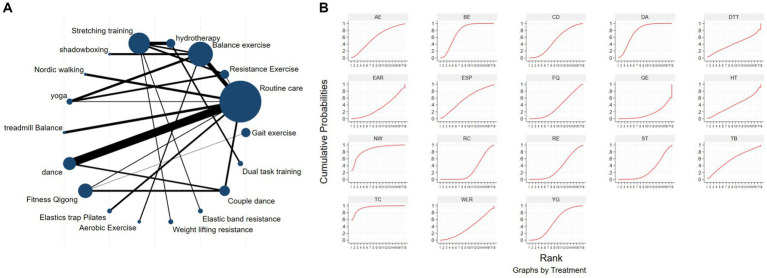
The NMA figure for BBS **(A)**. The SUCRA plot for BBS **(B)**. GE, gait exercise; RC, routine care; RE, resistance exercise; BE, balance exercise; HT, hydrotherapy; ST, stretching training; TC, shadowboxing; NW, Nordic walking; YG, yoga; TB, treadmill balance; DA, dance; FQ, fitness qigong; ESP, elastic strap pilates; AE, aerobic exercise; WLR, weight lifting resistance; EAR, elastic band resistance; CD, couple dance; DTT, dual task training.

The network diagram of different exercises on the BBS balance indicator in Parkinson’s disease is shown in Figure 6A, indicating that comparisons between DA (dance), ST (stretching training), BE (balance exercise), FQ (Fitness Qigong), and RC (Routine care) are common, and can also be considered as currently popular interventions for balance-related indicators in Parkinson’s disease. NMA was employed to compare the effects of different interventions on the balance ability of PD patients. In SUCRA (as shown in Figure 6B), different exercise interventions rank TC: shadowboxing (SUCRA = 94.7%) first in improving patients’ balance ability (increasing BBS scores). NW: Nordic walking (SUCRA = 86.7%), DA: Dance (SUCRA = 76.4%), BE: Balance exercise (SUCRA = 74.8%), AE: Aerobic Exercise (SUCRA = 59.3%), TB: treadmill Balance (SUCRA = 57.5%), ESP: Elastic strap Pilates (SUCRA = 56.8%), YG: Yoga (SUCRA = 53.4%), CD: Couple dance (SUCRA = 50.4%), DTT: Dual task training (SUCRA = 40.8%), HT: Hydrotherapy (SUCRA = 39.9%), WLR: Weight lifting resistance (SUCRA = 36.9%), FQ: Fitness Qigong (SUCRA = 36.3%) have BBS scores higher than the control group RC: (SUCRA = 27.4%). TC (shadow boxing) significantly outperforms the named control group (RC) (SMD = 11.22, CI = 3.26, 19.18). Additionally, TC exercise also significantly surpasses other currently popular interventions such as DA: Dance (SMD = 5.79, CI = −2.77, 14.36); BE: Balance exercise (SMD = −6.00, CI = −12.92, 0.92); RE: Resistance Exercise (SMD = −10.61, CI = −19.01, −2.22). The relative effect sizes of BBS efficacy are shown in Table 4. For consistency testing, a fitted inconsistency model yielded *p* > 0.05, indicating no significant inconsistency, confirming a consistency model. See Figure 5 for details.

**Table 4:** Relative effect sizes of efficacy on BBS, according to network meta-analysis.

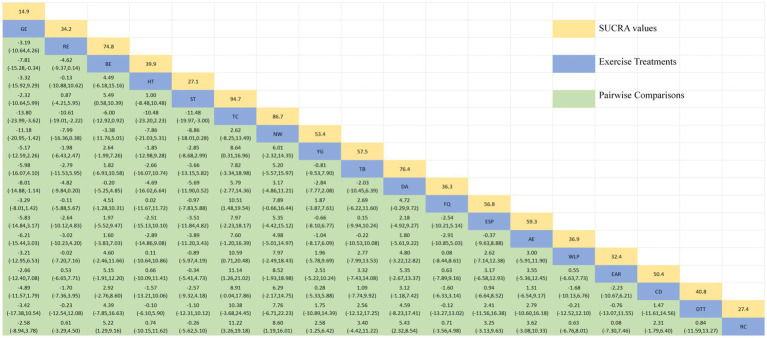

GE, gait exercise; RC, routine care; RE, resistance exercise; BE, balance exercise; HT, hydrotherapy; ST, stretching training; TC, shadowboxing; NW, Nordic walking; YG, yoga; TB, treadmill balance; DA, dance; FQ, fitness qigong; ESP, elastic strap pilates; AE, aerobic exercise; WLR, weight lifting resistance; EAR, elastic band resistance; CD, couple dance; DTT, dual task training. The green box is pair-to-pair comparison, the blue box is intervention name, and the yellow box is SUCRA values.

#### 3.4.4 Mini-balance evaluation systems test

**Figure 7:**
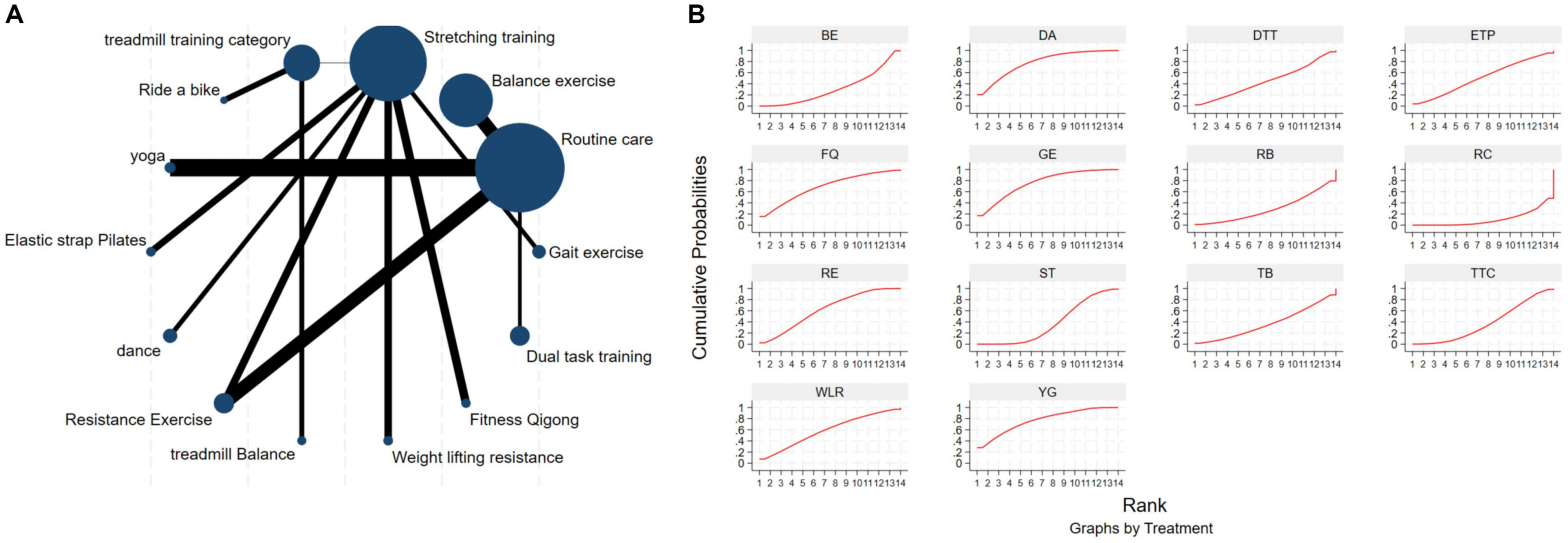
The NMA figure for Mini-BES Test **(A)**. The SUCRA plot for Mini-BES Test **(B)**. GE, gait exercise; RC, routine care; BE, balance exercise; ST, stretching training; TTC, treadmill training category; RAB, ride a bike; YG, yoga; ESP, elastic strap pilates; DA, dance; RE, resistance exercise; TB, treadmill balance; WLR, weight lifting resistance; FQ, fitness qigong; DTT, dual task training.

The NMA of the Mini-Balance Evaluation Systems Test (Mini-BESTest) is depicted in Figure 7A. In SUCRA, different exercise interventions are ranked in terms of the probability of increasing Mini-BESTest scores, with dance exercise ranking first, as shown in Figure 7B. Network meta-analysis results demonstrate that DA: Dance (SUCRA = 77.9%), YG: Yoga (SUCRA = 76.9%), GE: Gait exercise (SUCRA = 75.6%), FQ: Fitness Qigong (SUCRA = 68.5%), RE: Resistance Exercise (SUCRA = 62.3%), WLR: Weight lifting resistance (SUCRA = 58.7%), and ETP: Elastic strap Pilates (SUCRA = 51%) all show higher improvements in Mini-BESTest scores compared to the control group RC: Routine care (SUCRA = 10.6%). DA: Dance exercise significantly outperforms the control group (RC), with a potential effect estimate (SMD = 5.25, CI = −0.42, 10.92). Furthermore, DA demonstrates significantly better effects compared to other exercise interventions [TTC: treadmill training category (SMD = −2.9, CI = −7.15, 1.35), DTT: Dual task training (SMD = −2.55, CI = −3.89, 8.99), ST: Stretching training (SMD = −2.80, CI = −6.05, 0.45), TB: treadmill Balance (SMD = 3.1, CI = −2.26, 8.46), BE: Balance exercise (SMD = −3.53, CI = −9.41, 2.35), RB: Ride bike (SMD = −3.70, CI = −9.18, 1.78)]. The relative effect size of Mini-BESTest efficacy is shown in Table 5. For consistency testing, a fitted inconsistency model yielded *P* > *F* = 0.9091, indicating no significant inconsistency, confirming a consistency model. Furthermore, through node splitting, indirect comparison, and direct comparison, all *p*-values were greater than 0.05, indicating good model convergence and effective iteration of included studies. See Figure 5 for details.

**Table 5:** Relative effect sizes of efficacy on Mini-BES Test, according to network meta-analysis.

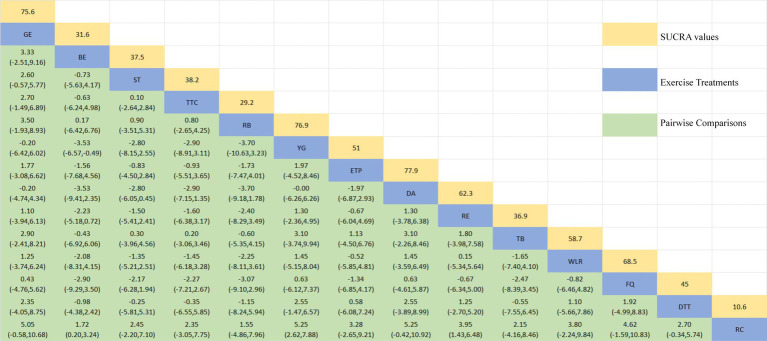

GE, gait exercise; RC, routine care; BE, balance exercise; ST, stretching training; TTC, treadmill training category; RAB, ride a bike; YG, yoga; ESP, elastic strap pilates; DA, dance; RF, resistance exercise; TB, treadmill balance; WLR, weight lifting resistance; FQ, fitness qigong; DTT, dual task training. The green box is pair-to-pair comparison, the blue box is intervention name, and the yellow box is SUCRA values.

#### 3.4.5 6-min walk test

**Figure 8:**
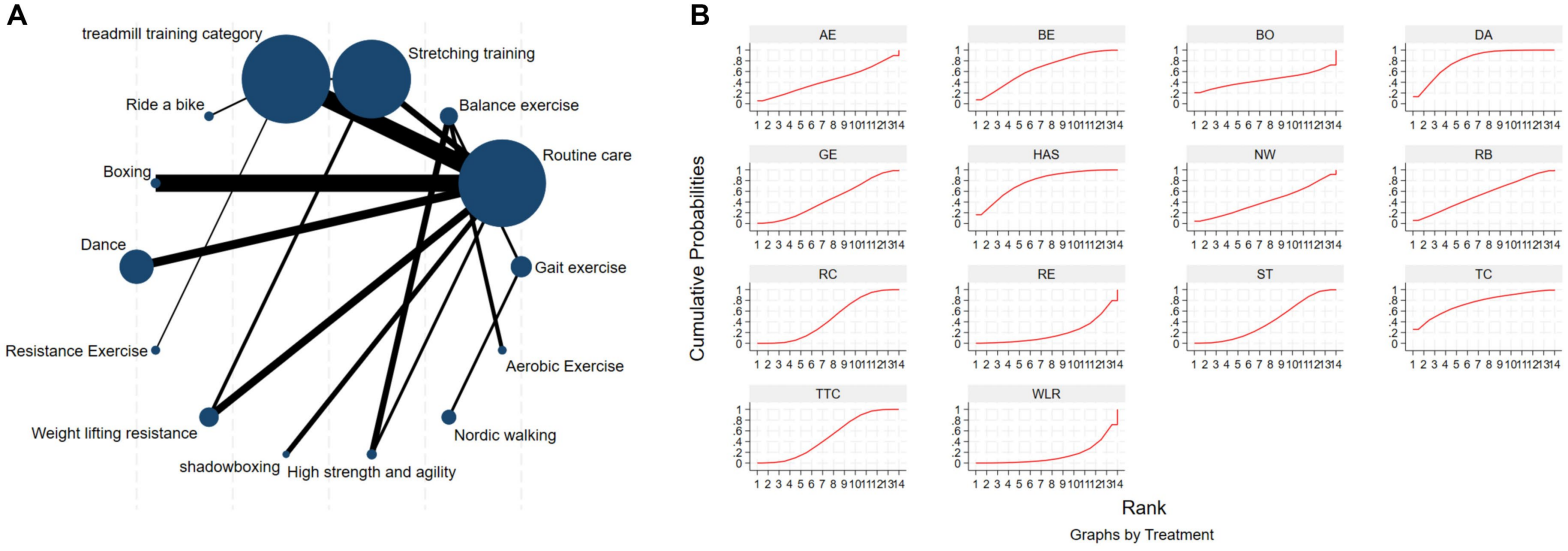
The NMA figure for 6MWT **(A)**. The SUCRA plot for 6MWT **(B)**. GE, gait exercise; RC, routine care; BE, balance exercise; ST, stretching training; TTC, treadmill training category; RAB, ride a bike; Bo, boxing; YG, yoga; DA, dance; RE, resistance exercise; WLR, weight lifting resistance; TC, shadowboxing; HAS, high strength and agility; NW, Nordic walking; AE, aerobic exercise.

The NMA of the 6-min walk test (6MWT) is depicted in Figure 8A. In SUCRA, different exercise interventions are ranked in terms of the probability of improving patients’ 6-min walking distance, with dance exercise ranking first, as shown in Figure 8B. Network meta-analysis results demonstrate that DA: Dance (SUCRA = 80.5%), HAS: High strength and agility (SUCRA = 77.0%), TC: Shadowboxing (SUCRA = 74.9%), BE: Balance exercise (SUCRA = 65.5%), RB: Ride bike (SUCRA = 54.4%), TTC: Treadmill training category (SUCRA = 49.1%), and RC: Routine care (SUCRA = 45.9%), GE: (SUCRA = 45.3%). DA: Dance exercise significantly outperforms the control group (RC) and gait training intervention (GE), providing potential effect estimates [RC (SMD = 71.31, CI = 13.77, 128.84); GE (SMD = −74.56, CI = −209.31, 60.20)]. Furthermore, DA demonstrates significantly better effects compared to other exercise interventions [ST: Stretching training (SMD = −80.11, CI = −172.92, 12.69); BO: Boing (SMD = −93.05, CI = −446.42, 260.32); RE: Resistance Exercise (SMD = 141.01, CI = 4.87, 277.14); WLR: Weight lifting resistance (SMD = 155.28, CI = 26.47, 284.08)]. The relative effect size of the 6-min walk test efficacy is shown in Table 6. For consistency testing, a fitted inconsistency model yielded *p* = 0.4090, indicating no significant inconsistency, confirming a consistency model. Furthermore, through node splitting, indirect comparison, and direct comparison, all *p*-values were greater than 0.05, indicating good model convergence and effective iteration of included studies. See Figure 5 for details.

**Table 6:** Relative effect sizes of efficacy on 6MWT, according to network meta-analysis.

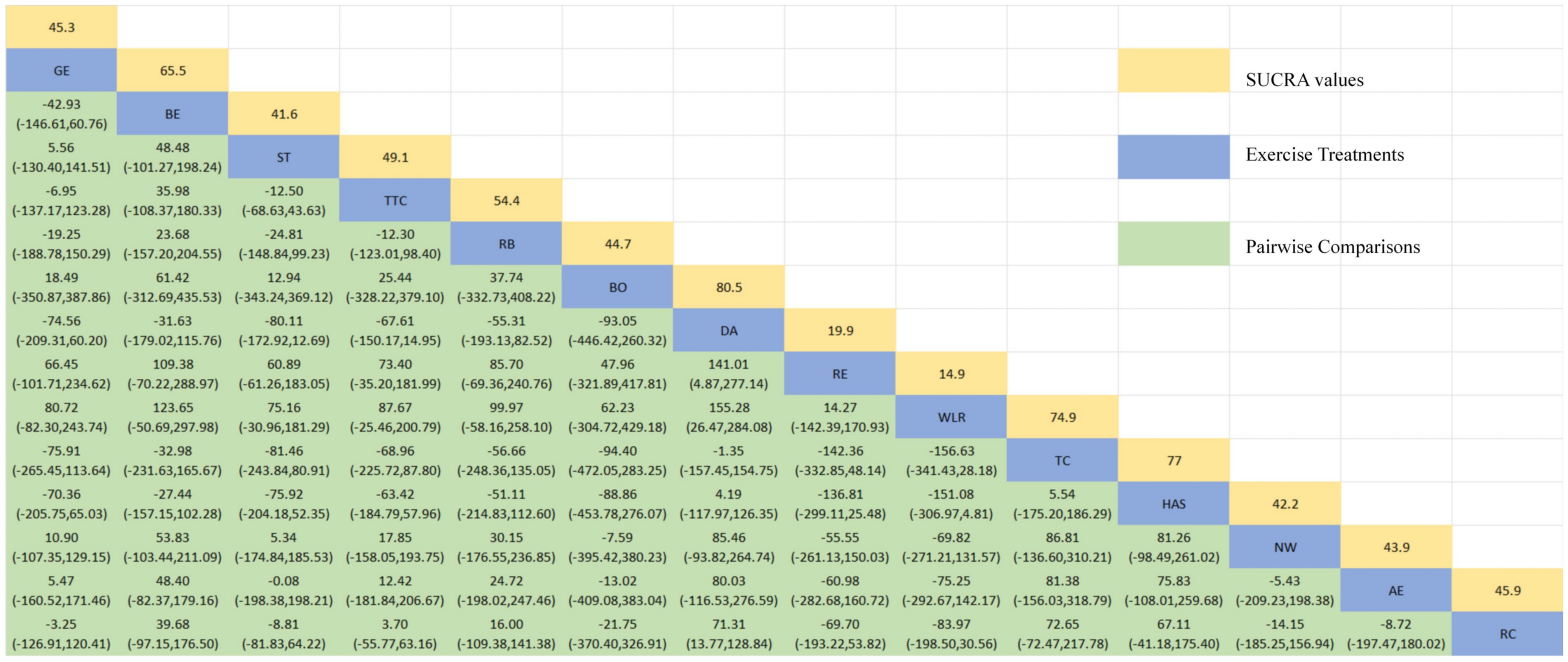

GE, gait exercise; RC, routine care; BE, balance exercise; ST, stretching training; TTC, treadmill training category; RAB, ride a bike; Bo, boxing; YG, yoga; DA, dance; RE, resistance exercise; WLR, weight lifting resistance; TC, shadowboxing; HAS, high strength and agility; NW, Nordic walking; AE, aerobic exercise. The green box is pair-to-pair comparison, the blue box is intervention name, and the yellow box is SUCRA values.

### 3.5 Publication bias test

Different funnel plots were created for all outcome measures in this study to examine potential publication bias. Visual inspection of the funnel plots did not reveal any significant publication bias. Figure 5 displays the funnel plots for MDS-UPDRS-III (Figure 5A), TUG (Figure 5B), 6MWT (Figure 5C), BBS (Figure 5D), Mini BES Test (Figure 5E), with all *p*-values >0.05.

## 4 Conclusion

This network meta-analysis and systematic review investigated the effectiveness of various exercise interventions on the mobility of Parkinson’s disease (PD) patients. A total of 111 studies were included, involving 5,358 participants, 133 intervention experiments, and 31 intervention measures. This study analyzed the improvement in mobility of PD patients through direct and indirect comparisons of different exercise interventions. The results showed that archery became the most effective intervention for reducing MDS-UPDRS-III scores and improving patient mobility (32, 61), while shadowboxing was the most effective intervention for improving BBS test scores in PD patients (44). High strength and agility training could significantly reduce the time taken by patients to perform TUG (91, 92), while dance was found to be the best exercise intervention for significantly improving Mini-BES Test scores and the walking distance in the 6MWT (68, 82, 94–96).

Of particular note, based on the comprehensive ranking of the five test results in this study, we suggest that slow-paced, balance-focused exercises such as tai chi, dance, and archery may be the most suitable exercise interventions for enhancing postural balance and mobility in PD patients. Conversely, faster-paced and higher-intensity exercises like high-intensity agility training may help patients with rapid movements, balance maintenance, and even aid in initiating movements to alleviate freezing of gait.

In terms of balance assessment, we selected the BBS and Mini-BES Test as two balance testing indicators. The former evaluates the ability to maintain safe balance in a series of predetermined tasks excluding gait assessment, while the latter, a simplified version of the Balance Evaluation Systems Test (BESTest), focuses solely on dynamic balance, making it more clinically applicable and able to differentiate between mild to moderate PD patients. The Time Up and Go test (TUG) is a simple and commonly used scale to assess the walking ability of the elderly (97). The 6MWT has been widely used to evaluate patients’ exercise tolerance, medical intervention effects, and disease prognosis, demonstrating good practicality and effectiveness. Finally, by combining these three tests with the MDS-UPDRS-III, this study synthesized them into a comprehensive indicator for assessing the mobility of PD patients.

As described above, tai chi, dance, archery, and high-intensity agility training are the most effective interventions for improving patient mobility (alleviating balance symptoms and improving walking ability), and I believe the underlying mechanisms behind these effects can be partially explained by two factors:

Firstly, targeted training can effectively improve the corresponding symptoms of patients. Compared to other exercise interventions, tai chi and dance are moderate-intensity aerobic exercises. In tai chi, movements involve diagonal motion (stepping out at a 45° angle from the body), which allows patients to stand with their feet apart and maintain balance without falling, thereby promoting lower limb muscle training and improving lower limb stability. Dance intervention, on the other hand, involves various components including balance maintenance, auditory, visual, and sensory reaction, memory, perception, expression, and social interaction (98), which may address stiffness, bradykinesia, and postural instability associated with PD (99–101), and also create a sense of pleasure through a combination of physical exercise and mental regulation, thereby improving compliance (102), stimulating the basal ganglia circuitry and reward system to evoke positive emotions (103). The rhythmic beats in dance and the rhythm of dance movements help improve patients’ freezing gait and enhance their mobility.

Archery is one of the earliest sports introduced into medical treatment for paralysis and quadriplegia. It is considered an ideal remedial exercise (104), activating the latissimus dorsi and serratus anterior muscles in the trunk. Additionally, it exercises the hand muscles involved in drawing the bow, all finger muscles, and wrist extension. When PD patients focus on drawing the bow, pulling the bowstring, twisting the hand, and releasing the arrow successfully, all the muscle groups involved are activated, thus offering the potential for strength enhancement. PD patients also exhibit a strong desire to hit the target during archery practice, which leads to continuous conscious control of their bodies. Therefore, continuous practice of archery can improve upper limb function, body stability, and elevate patients’ mood, effectively improving non-motor symptoms (32). However, there are not many randomized controlled trials (RCTs) currently using archery for intervention in Parkinson’s disease (PD), and a large number of RCTs are still needed to confirm these advantages.

Secondly, learning multiple skills and continuous practice can improve patients’ executive function and spatial perception, leading to significant changes in posture balance and mobility. These top-ranking exercise interventions are complex and demanding. Continuous practice can increase patients’ ability to allocate cognitive resources in time and space. For instance, high-intensity agility training was found to be the best exercise for the TUG index in this study. In this study, patients engaged in visual training: (1) quickly dodging simulated objects coming toward them, (2) performing extensive movements within a 6 square meter area to accurately target, and (3) accurately and quickly imitating the actions of companions on the screen. For PD patients, frequent external signal stimulation can increase striatal activity or promote the damaged basal ganglia-SMA loop to drive sensory-motor network activity, thereby improving patients’ mobility and compensating for the loss of dopaminergic stimulation. These benefits include helping PD patients create complex coordinated movement sequences and improving overall postural balance performance.

In fact, for PD patients, the preference or choice of exercise mode may not be particularly important, but long-term adherence to one exercise intervention, regardless of the type, may be more important than the choice of exercise mode itself.

### 4.1 Advantages and limitations

Firstly, this study is the most comprehensive and systematic comparative meta-analysis of the impact of exercise on the mobility of Parkinson’s disease (PD) patients. It included a total of 111 studies involving 5,358 participants, 133 intervention experiments, and 31 intervention measures. In contrast to other similar studies, we classified and refined exercise interventions. For example, we categorized interventions based on binary rhythm as one group, with partner dances (tango, waltz) as another, independent of the broader dance category (Irish dance, improvised dance). SUCRA proved that this decision was correct, providing us with a clearer understanding of exercise interventions to improve mobility in PD patients. In the analysis of the MDS-UPDRS-III indicator, Duality Rhythm Dance (SUCRA = 80.8%) ranked in the top three. This is a new discovery in this network meta-analysis (NMA) and a major highlight distinguishing it from previous NMAs. In previous studies, it was found through the analysis of previous randomized controlled trials that dance, dual-task training, rhythmic auditory training, and high-intensity resistance training significantly improved mobility in PD patients (105, 106). In the analysis of this NMA, these intervention measures also ranked highly, maintaining significant consistency with previous findings. However, previous reviews did not refine some distinctive exercise interventions, such as categorizing tai chi, fitness qigong, and boxing as one category, and categorizing various types of dances into one group (61). Although this classification yielded results, it still does not allow precise selection of appropriate interventions for patients and clinicians.

However, our study also has several limitations. Firstly, our NMA only included randomized controlled trials of PD patients with an average Hoehn-Yahr stage value of 1–3; therefore, the results of the NMA may not be generalizable to all PD patients. Secondly, there is heterogeneity in the frequency and duration of exercise interventions in the trials. Thirdly, although we comprehensively searched literature on exercise interventions for PD patients’ mobility, the language was limited to English, which may lead to selection bias. Fourthly, many comparisons of intervention measures only included a small number of trials, which may affect the accuracy of conclusions. Fifthly, the MDS-UPDRS-III score examines the severity of motor symptoms and disease progression in PD, with higher scores indicating more severe disease and progression. Although the people included in the analysis were classified as early to mid-stage (Hoehn-Yahr stage values of 1–3) PD, the MDS-UPDRS-III scores varied.

Finally, most studies did not report concealed allocation, which may lead to selection and performance bias. Therefore, these rankings have potential uncertainty and may not fully reflect reality. In the future, rigorously designed randomized controlled trials with larger sample sizes are needed to verify these findings.

## Data Availability

The original contributions presented in the study are included in the article/supplementary material, further inquiries can be directed to the corresponding author.

## Glossary

GE: Gait exercise
RC: Routine care
RE: Resistance Exercise
BE: Balance exercise
HT: hydrotherapy
AH: archery
ST: Stretching training
TC: shadowboxing
NW: Nordic walking
YG: yoga
TTC: treadmill training category
TB: treadmill Balance
DA: dance
SG: Sports game
MM: Mindfulness meditation
DTT: Dual task training
FQ: Fitness Qigong
HAS: High strength and agility
SC: Sport climbing
SST: Sports strategy training
DA2: dance2
BO: Boxing
MIT: Moderate intensity training
STT: Sensory attention training
ESP: Elastic strap Pilates
RAB: Ride a bike
AE: Aerobic Exercise
WLR: Weight lifting resistance
EAR: Elastic band resistance
CD: Couple dance
BK: Kick boxing
